# Temporal attention causes systematic biases in visual confidence

**DOI:** 10.1038/s41598-019-48063-x

**Published:** 2019-08-12

**Authors:** Samuel Recht, Pascal Mamassian, Vincent de Gardelle

**Affiliations:** 1 Laboratoire des systèmes perceptifs, Département d’études cognitives, École normale supérieure, PSL University, CNRS, 75005 Paris, France; 2 CNRS and Paris School of Economics, Paris, France

**Keywords:** Attention, Perception, Human behaviour

## Abstract

Temporal attention enhances the perceptual representation of a stimulus at a particular point in time. The number of possible attentional episodes in a given period is limited, but whether observers’ confidence reflects such limitations is still unclear. To investigate this issue, we adapted an “Attentional Blink” paradigm, presenting observers with a rapid visual stream of letters containing two targets cued for subsequent perceptual reports and confidence judgments. We found three main results. First, when two targets fell within the same attentional episode, the second target underwent a strong under-confidence bias. In other words, confidence neglected that a single attentional episode can benefit to both targets. Second, despite this initial bias, confidence was strongly correlated with response probability. Third, as confidence was yoked to the evidence used in perceptual reports, it remains blind to delays in response selection for the second target. Notably, the second target was often mistaken with a later item associated with higher confidence. These results suggest that confidence does not perfectly evaluate the limits of temporal attention in challenging situations.

## Introduction

Visual confidence is the subjective estimation of the accuracy of a decision made about a visual stimulus^1^. It typically correlates with the objective accuracy of the decision, and can be used to regulate behavior^2–4^. However, humans do not always monitor their performance perfectly, and dissociations between confidence and performance have been documented^5–9^. Here, our goal is to assess how observers’ confidence and performance are affected when temporal attention is challenged, and whether confidence tracks the limits of temporal attention.

Temporal attention enhances a stimulus at a particular point in time^10^ and inhibits other time points^11^, much like spatial attention does in space^12^. Both attention and confidence are related to accuracy: attention increases the signal-to-noise ratio of the stimulus, while confidence ideally reflects this increase. Attention and confidence have already been studied together in the spatial domain, leading to mixed findings: some studies observed a dissociation between the two^6,13,14^, while others suggested that spatial attention is well incorporated into confidence^15–18^. In the time domain, this link between temporal attention and confidence remains largely unexplored. This question is particularly relevant given the possibility that attention and confidence might operate at different time scales^19^.

In some circumstances, temporal attention can be suppressed, delayed or misplaced. One robust finding regarding the limits of temporal attention is the “Attentional Blink”^20,21^. Specifically, when two targets are embedded in a rapid serial visual presentation stream, the second target T2 is often missed when it appears soon (150–300 ms) after the first target T1. When temporal selection is not simply suppressed in the case of missed T2 targets, it is delayed, such that an item following T2 would be reported instead. These selection delays, sometimes known as “post-target error intrusions”^22,23^ are a second feature of the Attentional Blink. Finally, when T2 is presented immediately after T1 (60–100 ms), then both targets are on average accurately reported. This effect, coined the “lag-1 sparing”^24^ is a third feature of the Attentional Blink. These three features can be accounted for by a variety of models^25,26^. However, whether confidence tracks these three features remains an open empirical question.

To address this question, we used an Attentional Blink paradigm in combination with confidence judgments, in order to evaluate whether participants’ confidence judgments about T2 reports would reflect the suppression of accuracy during the Attentional Blink, the sparing of accuracy at lag-1, and the delay in temporal selection that follows the Attentional Blink. We also collected confidence judgments for T1 as a comparison baseline. To measure errors and delays in temporal selection, we presented participants with a rapid stream of letters, and indicated two letters in the stream for later report. The serial position of each letter in the stream provided critical information on the point in time at which attention was deployed^27–29^. In other words, the present work proposes to investigate whether participants accurately evaluate the limits of their ability to deploy their attention at the right moment in time.

## Material and Methods

### Participants

39 adult volunteers were recruited from the Laboratoire d’Economie Expérimentale de Paris (LEEP) pool of participants (M ± SD = 25.5 ± 2.9 years old, 17 females). They all provided informed written consent prior to the experiment. The sample size was based on a recent study involving a highly similar Attentional Blink paradigm^27^. The present experiment was also replicated with a similar sample size (see Experiment 2 in Supplementary Material). Four observers were discarded because of a technical problem, and three participants were removed because of extremely low accuracy rate for target 1 or 2 (exclusion criterion: <10% accuracy), leaving 32 participants for analysis. Observers were paid a base sum (10 EUR) plus a bonus depending on their performance in the task (up to 10 EUR in addition). The average payoff was 16.43 EUR (SD = 1.89) for a single 1.5 hours session. The experimental procedure received approval from the Paris School of Economics (PSE) ethics review board and adhered to the principles of the Declaration of Helsinki.

### Apparatus and stimuli

**Figure 1:**
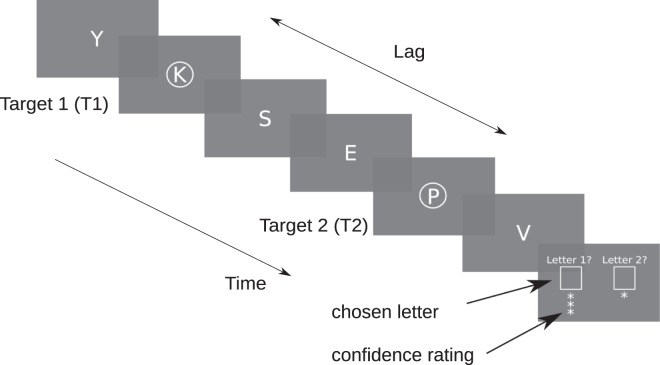
Experiment design. Participants were required to report the two cued letters in the RSVP, and rate their confidence for each reported letter (Experiment 1) or for only one of the letter (Experiment 2, see Supplementary Material) on a three-point scale. The distance in items (or lag) between the first target (T1) and second target (T2) was varied across trials (lag-3 depicted here). Each letter appeared for 33 ms, followed by a 50 ms ISI.

Participants sat approximately 60 cm from the screen (1280 × 1024 pixels, 60 Hz refresh rate). Stimuli were generated using the Python programming language and the PsychoPy toolbox^30^ on a Windows XP computer. On each trial, participants were presented with a rapid serial visual presentation (RSVP) stream of the 26 English letters (Courier New, white font, 2.5° of visual angle) in the center of a black screen background (Fig. 1). Letters were randomized, and each letter was presented for 33 ms (2 frames) with an inter-stimulus interval of 50 ms (3 frames). Two letters in the stream were targets surrounded by a visual cue (white annulus, inner/outer diameter: 2.9°/3.1°), which appeared simultaneously with the target. The first target (T1) was located between the 5th and the 10th item in the stream, while the second target occurred at the 1st, 2nd, 3rd, 6th or 9th position after T1. Both target positions where counterbalanced with a full factorial design.

The lags between T1 and T2 were chosen in order to sample the different periods of the Attentional Blink: lag-1 (83 ms after T1), where lag-1 sparing is known to occur; lags 2 and 3 (166 ms and 249 ms), which usually show strong drop in T2 reporting accuracy; and finally lags 6 through 9 (498 ms and 747 ms) that demonstrate a progressive recovery in accuracy.

### Procedure

At the end of each trial, participants had to report each target letter, in order of appearance, as well as their confidence for each report, using a French keyboard. Duplicates of the same letter were not accepted, given that each letter only appeared once in the stream. Confidence ratings were given on a 3-point scale using the numerical pad. For T1 confidence, keys 1, 4 and 7 corresponded to low, medium and high confidence. For T2 confidence, keys 3, 6 and 9 corresponded to low, medium, and high confidence. The confidence rating given to each target was displayed as one to three stars appearing below each of the reported letters. Participants could correct their response and confidence as needed. Participants validated their responses by pressing the Shift key.

Confidence was also incentivized. Specifically, participants were informed that each of their responses would generate 1, 2 or 3 points depending on their confidence rating. Points will be considered “good” if the response is correct and worth 0.5 EUR, and “bad” for incorrect responses and worth 0 EUR. Every 25 trials, the computer would randomly draw one point from those generated by the participant in the past 25 trials. The randomly drawn point, which could be “good” or “bad”, determines the reward for these 25 of trials. This approach was applied separately to T1 and T2 responses. At the end of the experiment, the sum of these draws was used to estimate the monetary reward of the participant. The goal of this procedure was to engage participants in using confidence rating scale as accurately as possible during the whole experiment. High accuracy and good confidence estimates were therefore decisive to maximize payoff. Participants did not receive accuracy feedback until the very end of the experiment.

Before the main experiment, participants completed 10 practice trials, the first half without confidence judgments. The main session then consisted in 500 trials, with a 10-seconds break every 60 trials.

### Analyses

All the analyses were carried out using the R programming language. Mixed effects models were built using the Lme4 R package. Accuracy and average confidence of T1 and T2 reports were analyzed using standard ANOVAs. In the current paradigm, the position of the reported item is also of interest. To analyze how reports and confidence depended on this serial position, a mixed effects model comparison approach was used. Specifically, a regression with fixed effects of position (and possibly other factors) and participants as random intercepts was compared to a regression without the fixed effect of position. When necessary, a third model including an interaction was added to the comparison.

Statistical results involving serial positions were systematically confirmed using permutation analysis, given the unbalanced nature of the dataset in this case. Serial positions were randomly shuffled for each participant and lag separately (for the whole dataset) and the relevant statistical analysis was applied to these surrogates data. The process was repeated 3,000 times, and the resulting distribution was compared to the test result obtained on the original data. P-values obtained through this method are reported as ‘p_RAND_’.

When necessary, ANOVAs were corrected using the Greenhouse-Geisser adjustment and t-tests were corrected using the Welch-Satterthwaite adjustment. We report Wilcoxon signed ranked test using uppercase T when the Shapiro-Wilk normality test failed, and Student test using lowercase t otherwise.

## Results

### Overview

We start our result section by focusing on the first target (T1), which constitutes a baseline to evaluate how confidence is linked to reports when attention is unchallenged. In brief, for T1 we found that reports were distributed around the true position, and that confidence for these reports decreased with the distance to the target, following a bell-shaped profile similar to the one seen in report probability.

We then turn to our main results, which concern the second target (T2), known to be affected by the Attentional Blink. There are three main findings. First, both confidence and accuracy drop at lag-2 and lag-3, and confidence failed to reflect the sparing of accuracy at lag-1. Second, confidence was strongly correlated with the frequency of item selection (as was found for T1). A simple model for this correlation will be detailed in the discussion and simulations for this model can be found in the Supplementary Material. Our third result is that confidence was oblivious to the delays in item selection: after the Attentional Blink and up to lag-9, reports were systematically delayed relative to the target, and confidence was also shifted towards delayed responses, consistently with the correlation between confidence and frequency.

### T1: Probability of report and confidence are strongly correlated

**Figure 2:**
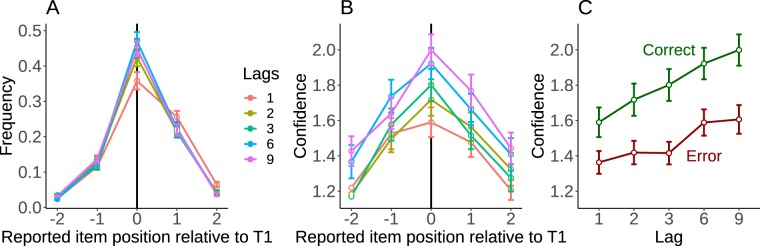
Reports and confidence for the first target. (**A**) The frequency of reports for item around target true position, separately for each lag. (**B**) The average confidence per position, for each lag. (**C**) The average confidence level for correct responses and errors, which provides an estimate of metacognition. Error bars represent standard error of the mean across participants.

Overall, T1 targets were identified correctly 43% of the time. As can be seen on Fig. 2A, and as documented previously^29^, errors were not random guesses. The letter presented just before or just after the target was reported in 18% of the trials, largely exceeding the guess rate of 1/26 ≈ 4% (t(31) = 21.7 p < 0.001). Focusing on the 5 serial positions around T1 (included), we further tested how report frequency can be predicted from the position, the lag and their interaction (using mixed models, see Analyses). Including item position as a predictor outperformed a model without the position effect (χ^2^(4) = 1058, p_RAND_ < 0.001). Including the lag × position interaction improved the model even further (χ^2^(16) = 43.3, p_RAND_ = 0.003), but this interaction seemed specifically driven by the lag-1 as it disappeared when excluding this lag from the analysis (χ^2^(16) = 5.6, p_RAND_ = 0.95). The interaction between lag and position might reflect the confusion and order reversals that occur at lag-1 (see Supplementary Material).

One striking feature of the data is that confidence followed a profile similar to report frequency: when a specific position was reported more frequently, these reports were also associated with greater confidence (Fig. 2B). Confidence was significantly affected by item position (χ^2^(4) = 240, p_RAND_ < 0.001). Including the interaction between lag and position however did not improve the model (χ^2^(16) = 15.8, p_RAND_ = 0.48). We replicated these analyses while excluding correct responses, to confirm that these results did not merely reflect the ability to discriminate between correct and erroneous responses.

To directly evaluate the similarity between confidence and report frequency, confidence was averaged for each participant by grouping all lags together, and we correlated this average confidence to the report frequency, across the 5 report positions centered on the target (including the target’s position). The mean r coefficient was 0.86, across participants (95% CI = [0.82 0.90]; t(31) = 44.2, p_RAND_ < 0.001). Thus, it appears that participants’ confidence is closely linked to the probability with which the reported letter is selected.

One typical signature of metacognition is the difference of confidence between correct and incorrect reports, with higher confidence for correct responses. Figure 2C illustrates this measure for the different lags. A repeated-measures ANOVA with lag and trial type (correct vs. error) revealed a main effect of trial type (F(1,31) = 77.8, MSE = 0.11, p < 0.001), a main effect of lag (F(2.04,63.4) = 38.2, MSE = 0.06, p < 0.001), as well as a lag × type interaction (F(3.35,104) = 5.7, MSE = 0.02, p < 0.001). Overall, participants gave higher confidence to correct than to incorrect T1 responses. This difference between trial types increased with the lag between T1 and T2, but was present for all lags (all p < 0.01, alpha = 0.05/5, Bonferroni-corrected for 5 comparisons).

### T2: Confidence tracks the Attentional Blink but not Lag-1 sparing

**Figure 3:**
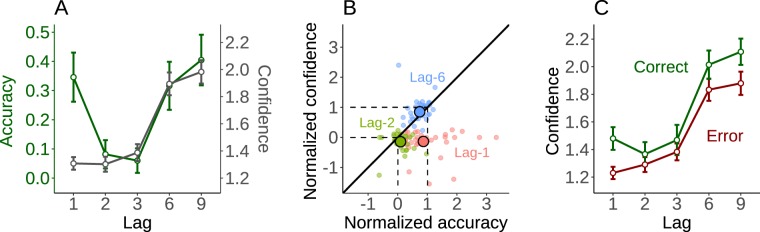
Attentional Blink and early confidence bias. (**A**) T2 average accuracy (in green) and confidence (in grey) as a function of the lag between T1 and T2. (**B**) The systematic under-confidence occurring at lag-1 (83 ms after the first target) is illustrated by representing accuracy and confidence for lag-1 (in red) in the space from lag-3 to lag-9. The dashed lines represent (0, 0) coordinates corresponding to lag-3 and (1, 1) coordinates corresponding to lag-9 in this space. As a comparison, lag-2 (in green) and lag-6 (in blue) are pictured as well. Each colored point is a participant in the considered condition. The means for each condition are black-circled. Points below the diagonal represent under-confidence. (**C**) The average confidence level for correct T2 reports and errors, for each lag. Metacognitive sensitivity is conserved at lag-1 despite a bias for low confidence ratings. Error bars represent standard error of the mean across participants.

We then analyzed reports and confidence judgment about T2 targets (see Fig. 3A). To make sure of a successful initial attentional capture by T1, we analyzed only trials in which T1 was correctly reported. In these trials, 23% of T2 reports were correct. Figure 3A shows T2 accuracy and confidence for the different T1-T2 lags. T2 accuracy was affected by the T1-T2 lag (F(2.14,66.5) = 67.2, MSE = 0.02, p < 0.001) and exhibited the classical Attentional Blink effect: it dropped for lag-2 and lag-3 relative to longer lags (2–3 vs. 6–9: T(31) = 0, p < 0.001). Confidence was also affected by lag (F(1.88,58.4) = 92.4, MSE = 0.08, p < 0.001) and dropped for lags 2–3 relative to longer lags (2–3 vs. 6–9: T(31) = 0, p < 0.001), paralleling accuracy. Thus, participants were able to acknowledge the drop of performance at lags 2–3 relative to longer lags.

Importantly however, participants’ confidence was strongly dissociated from accuracy at lag-1. Confidence seemed blind to lag-1 sparing, a classical phenomenon where T2 accuracy at lag-1 is much higher than during the blink period (1 vs. 2–3: T(31) = 528, p < 0.001) and indistinguishable from long lags (1 vs. 6–9: T(31) = 260, p = 0.95). Indeed, lag-1 confidence was as low as for lag 2–3 (T(31) = 197, p = 0.66) and much lower than for long lags (1 vs. 6–9: T(31) = 0, p < 0.001).

To further quantify this “lag-1 under-confidence”, we asked whether the increase in accuracy at lag-1 relative to lag-3 was accompanied by the corresponding increase in confidence. Specifically, for each participant we regressed confidence against accuracy using lag-3 and lag-9 average data. The predicted confidence at lag-1 was then interpolated from the accuracy at lag-1, using this regression. Across participants, the observed confidence was significantly lower than the predicted confidence level (M = 0.63, 95% CI = [0.45 0.81]; t(31) = 7.1, p < 0.001, alpha = 0.05/3). For comparison, we also applied this approach to lag-2 and lag-6. Some under-confidence was found for lag-2 (M = 0.14, 95% CI = [0.07 0.21]; t(31) = 3.9, p < 0.001, alpha = 0.05/3). For lag-6 we found no difference between predicted and observed confidence (M = −0.07, 95% CI = [−0.13 0.003]; t(31) = −1.9, p = 0.06, alpha = 0.05/3).

Figure 3B illustrates this analysis by plotting confidence against accuracy, in the lag-3-to-9 space. For each participant, normalized accuracy was calculated as (x_1_−x_3_)/(x_9_−x_3_), where x_k_ is the accuracy at lag-k, and the same procedure was done for confidence. For lag-1, all participants are located below the diagonal, suggesting that they are less confident than what could be expected given their accuracy. Figure  3B further illustrates how lag-6 and lag-1 differ in terms of confidence but not in terms of accuracy, whereas lag-2 and lag-1 differ in terms of accuracy but not in terms of confidence.

We then focused on metacognition, defined above as the difference in confidence between correct reports and errors. Because some participants had no correct answers at lag-2, only a subset of participants was considered here (N = 25). As can be seen from Fig. 3C, participants overall expressed higher confidence when they were correct and higher confidence at longer lags. A repeated-measures ANOVA with lag and trial type (correct vs. error) confirmed these two main effects (error vs correct: F(1,24) = 11, MSE = 0.15, p = 0.002; lag: F(2.37,56.92) = 58.5, MSE = 0.15, p < 0.001) and indicated an interaction (F(3.46,83.1) = 3.28 MSE = 0.05, p = 0.02). Post-hoc Bonferroni-corrected tests (alpha = 0.05/5) showed that the difference in confidence between correct reports and errors was significant for lag-1 (t(24) = 3.7, p = 0.001), lag-6 (t(24) = 3.1, p = 0.004) and lag-9 (t(24) = 4.3, p < 0.001) but not for lag-2 (t(24) = 1.4, p = 0.18) or lag-3 (t(24) = 0.1, p = 0.89). In other words, the ability to detect objective errors was diminished specifically during the Attentional Blink period. Note that this is not surprising given the well-known relation between metacognitive sensitivity and task performance^31^. Interestingly, it did not disappear at lag-1, despite the low level of confidence.

### T2: probability of report and confidence are strongly correlated

**Figure 4:**
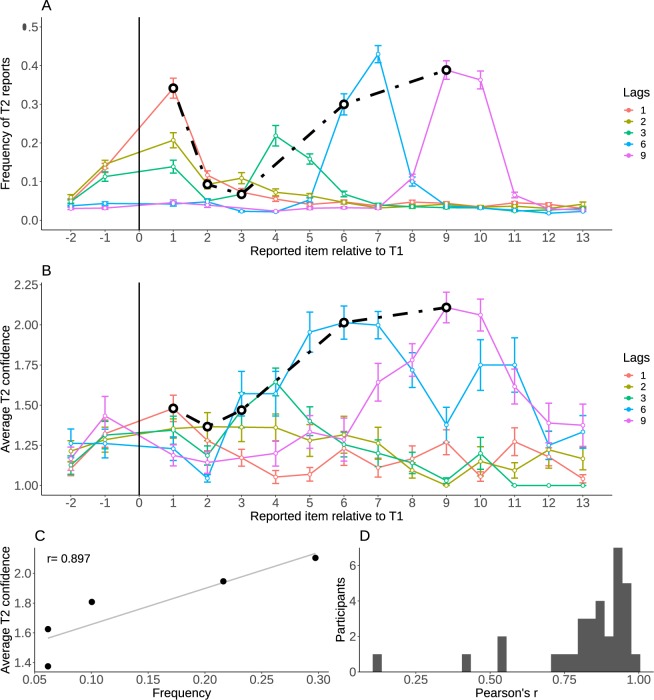
Reports and confidence for the second target. (**A**) The frequency of T2 reports as a function of the position of the reported item relative to T1, for each lag. Note that T1 position has no value, given that only trials in which T1 is correctly reported were considered here (hence T2 reports cannot correspond to T1 position). The black line connects the points corresponding to accurate T2 reports. (**B**) Confidence of the T2 reports, as a function of the position of the reported item relative to T1, for each lag. The black line connects the points corresponding to accurate T2 reports. Error bars represent standard error of the mean across participants. (**C**) Regression between frequency and confidence with 5 positions centered on T2, collapsed across lags, for a representative participant. (**D**) Histogram of the correlation coefficients for all the participants. The confidence-frequency relation is strong and holds for most participants.

Similarly to T1, errors for T2 reports were not random guesses but distributed around the correct target position. In particular, items appearing just before or just after the target were reported more often than chance (17%, with a 95% CI = [0.16 0.18]; vs. chance level at 4%: t(31) = 19.5, p < 0.001). Comparing Fig. 4A,B, we note that for each lag confidence and report frequency typically peak at the same item position, even when this item position is not the target position. This similarity between confidence and report frequency across positions was examined for each individual participant, by considering 5 positions centered on T2, after averaging across lags. Figure 4C shows a representative participant and Fig. 4D shows the distribution of correlation coefficients at the group level, which confirm the strong relation between confidence and report frequency (Mean r coefficient: 0.82, 95% CI = [0.76 0.89]; t(31) = 25.3, p_RAND_ < 0.001). A correlation between confidence and log-frequency provided equivalent results.

### T2: Confidence does not correct for attentional delay

**Figure 5:**
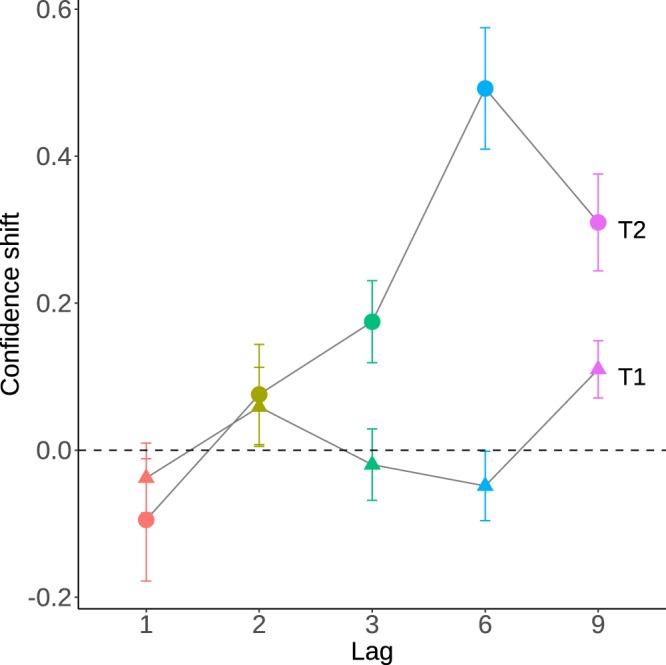
Confidence for T2 is delayed. Confidence shift is the average confidence in post-target minus pre-target errors, evaluated separately for each lag and for T1 (triangles) and T2 (dots). A positive value corresponds to greater confidence for post-target errors, that is, a shift of the confidence peak towards more delayed items. T2 confidence is delayed for lags 3, 6 and 9, reproducing the delay generally observed in items selection after the Attentional Blink period (see Fig. 4A and Supplementary Material). Error bars represent standard error of the mean across participants.

Attention is typically delayed after the Attentional Blink, as participants tend to report items that follow the target rather than the target itself. To analyze the delay in selection and confidence induced by the reorienting of attention (T2), we calculated the average position of the reported item relative to the target position, in an 11-items window centered on the target position. This measure, called the “center of mass” was positive for lags 6 and 9, showing that a delay occurred in item selection, as found in previous studies^23,27,29^ (see Supplementary Material). Given that confidence was correlated with report frequency, we investigated whether confidence was similarly shifted towards delayed selections. To do so, we calculated the average confidence for reports corresponding to late selections (“post-target” errors) minus the average confidence for early selections (“pre-target” errors). This “confidence shift” (Fig. 5) was evaluated over an 11-items window centered on (but excluding) the target position, separately for each lag. A model comparison approach confirmed that including the pre-target/post-target factor as a predictor for average confidence significantly outperformed the null model (χ^2^(1) = 27.1, p_RAND_ < 0.001). The interaction between lag and shift was also significant (χ^2^(4) = 34.8, p_RAND_ < 0.001). T-tests (Bonferroni-corrected for 5 lags with alpha = 0.05/5) confirmed a significant delay for lag-3 (t(31) = 3.13, p = 0.004), lag-6 (T(31) = 406, p < 0.001) and lag-9 (T(31) = 354, p < 0.001) but not for lag-1 and lag-2 (all p > 0.3). For comparison, this analysis showed no confidence shift when applied to T1 (χ^2^(1) = 0.3, p_RAND_ = 0.5).

### A replication with a reduced metacognitive load (experiment 2)

In Experiment 1, participants reported their confidence for both T1 and T2 targets in each trial. The high demand put on the metacognitive system during the task might explain why confidence failed to track the lag-1 sparing or the delays in item selection induced by the Attentional Blink. To address this possibility, we conducted a second experiment in which we lowered the demands put on the metacognitive system, by asking only one confidence estimate per trial. In experiment 2, participants (N = 29) gave their confidence about T1 in the first half of the experiment and their confidence about T2 in the other half (or vice-versa, counterbalanced across participants). All other parameters were identical to Experiment 1, and performance levels in Experiment 2 were similar to Experiment 1, with an average accuracy at 40% for T1 and at 22% for T2 after a correct T1 response (see Supplementary Fig. S1 and S2).

Critically, in Experiment 2 we replicated the three main findings of Experiment 1, as summarized below (for details see the Supplementary Material). First, participants were oblivious to lag-1 sparing and exhibited a clear under-confidence at lag-1 for their T2 reports (see Supplementary Fig. S3). Second, we replicated the finding that confidence was tied to report frequency for T1 (Supplementary Fig. S4). Hence, when a particular item was more likely to be selected, it was also reported with a greater confidence. Finally, both temporal selection and confidence were delayed after the Attentional Blink (Supplementary Fig. S5). In other words, whereas the metacognitive task was less demanding, participants were not better at acknowledging the lag-1 sparing or delays in temporal selection induced by the Attentional Blink.

## Discussion

The present study considered how human observers could evaluate their own performance in a task in which temporal attention has to be oriented towards two targets (T1 and T2) presented in close succession. To do so, confidence judgments were introduced within an Attentional Blink paradigm, and we analyzed how such judgments would track the limits of performance typically observed in this paradigm. We obtained three main results. First, participants failed to notice the early sparing of accuracy at lag-1, despite being able to detect the drop of accuracy at lag-2 and lag-3. Second, participants’ confidence when reporting an item systematically followed the probability of selecting this item in the sequence. Third, and likely because of this confidence-probability coupling, participants were oblivious to the delays in temporal selection induced by the Attentional Blink. All these results were replicated in a second experiment in which we only collected one confidence judgment (either for T1 or for T2), to reduce the demands put on the metacognitive system.

### Confidence is blind to lag-1 sparing

Surprisingly, confidence was not able to track the sparing of accuracy known to occur when the two targets are very close in time. However, we note that metacognition was not particularly altered during lag-1: participants still discriminated between correct responses and errors, and between different errors (Fig. 3). This under-confidence is therefore not due to participants being unable to use their metacognition. Nonetheless, confidence did not adjust to lag-1 sparing, despite its ability to track the drop in accuracy during lag-2 and lag-3, and the progressive recovery for longer lags. A confidence cost was systematically applied to all responses for lag-1, and this early under-confidence bias was present for almost every participant.

One possibility is that the under-confidence bias at lag-1 results from participants being aware of possible order reversals, where T1 would be reported as T2 and vice-versa due to temporal selection uncertainty (see Supplementary Material). Order reversals have been documented in the literature, and it has been suggested that at lag-1, T2 would actually benefit from the T1 attentional episode, the two targets being often perceived as a single object^24,27,32^, at the cost of an increased uncertainty about their relative order. This increased uncertainty could lead participants to express lower confidence.

Our confidence data at lag-1 seem to mirror what was found for visibility in a recent study that suggested lower visibility despite high accuracy^33^ at lag-1. However, another study^34^ found that subjective visibility during lag-1 is spared. Besides these mixed findings for visibility, one might consider that confidence and visibility do not always go hand-in-hand, and can be dissociated both conceptually and empirically^35,36^.

### A simple model of the confidence-frequency relation

The second major result of our study is that confidence generally follows report frequency across the items in the sequence. This robust correlation was observed on both T1 and T2, and irrespectively of the T1-T2 lag or the delays induced by the Attentional Blink. This finding speaks to the ongoing debate regarding whether the same evidence signal is used for decisions and confidence, and the observed dissociations between confidence and accuracy^5–9^. In our study, the under-confidence at lag 1 illustrates such a dissociation, but seems to exist on top of the strong relation between confidence and reports, suggesting that decisions and confidence judgments are also relying on the same evidence signal^37,38^.

The robust confidence-frequency relation found in the present work could be well accounted for by a simple attentional selection mechanism within a RSVP stream, based on the Attentional Gating Model^39^. In this model, the letters presented in the RSVP stream lead to a short-lasting activation of the corresponding letter-detectors in the perceptual system. When the cue appears, it triggers an attentional boost that enhances the response of the letter-detectors. This boost is smoothly distributed in time over several items. At the end of the sequence, the evidence for each item is the integral of the activity of the corresponding letter-detector, corrupted by random perturbations (i.e. noise). The item selected for report will be the one with maximum evidence. In fact, under the simple assumption that confidence relates to the amplitude of this evidence, a correlation between confidence and report frequency would occur across trials. To understand why, note that noise on evidence levels would move the peak evidence away from the correct target, thereby producing errors distributed around the target. These perturbations would also affect the confidence in these reports. Simulations of this process produced a correlation between confidence and report frequency across positions, as was found in our data. Details of this model are presented in the Supplementary Material (see Supplementary Fig. S6 – S10).

This proposed model accounts for (i) the correlation between report confidence and report frequency, (ii) the related observation that confidence is higher for correct responses than for errors, (iii) the finding that this metacognition is present mostly outside of the Attentional Blink and (iv) the result that confidence was blind to selection delays. However, it is important to highlight that this mechanism linking confidence and reports does not account for the under-confidence at lag-1. We believe that accommodating this last result would require additional components. Incorporating this mechanism within a full computational model of the Attentional Blink is a task for future research.

### Confidence does not correct for attentional delay

Our last result relates to the delayed attentional selection induced by the Attentional Blink. We found for both experiments a long-lasting delay in selection after the Attentional Blink, at lag-6 and lag-9, replicating previous findings^22,23^. Confidence remained fully oblivious to this fundamental limitation of the attentional system, an expected result given the correlation found between confidence and report frequency (Fig. 4D).

There is a striking similarity between the present finding about confidence in the Attentional Blink paradigm and a finding about introspective response times in the Psychological Refractory Period paradigm^40,41^. In this paradigm, two tasks have to be conducted in short succession in time, and the decision process for the second task is postponed until the first decision process has been completed. Interestingly however, introspective estimates of response times are blind to this delay. It has been suggested that the Attentional Blink and Psychological Refractory Period paradigm involve a similar central bottleneck^42,43^. Indeed, introspective measures of performance (respectively, confidence and subjective estimates of response times) appear to be oblivious to the delays presumably induced by this central bottleneck in both paradigms. To expand this research, future work might investigate whether introspection is blind to central delays in different paradigms, or to other constraints of central processing stages (e.g. the discrete/symbolic nature of information processing at central stages^44,45^).

## Conclusion

The strong correlation between frequency of reports and confidence during temporal selection (T1), which holds when attention has to reorient to a second point in time (T2), suggests that decision and confidence are mostly sharing the same evidence signal during the temporal orienting of attention. This tight coupling might prevent confidence from accessing delays in selection induced by the Attentional Blink, as shown in the present work. In addition, confidence seems to be affected by a heuristic penalizing a target that is too close in time from a prior attentional episode, a penalty that would account for the lag-1 under-confidence. These multiple phenomena suggest that confidence does not perfectly evaluate the state of temporal attention in challenging situations, likely because of late heuristic bias and the fact that confidence is yoked in time to temporal attention.

## Supplementary information

Supplementary Material

## Data Availability

Data for both experiments have been made publicly available via Open Science Framework and can be accessed at https://osf.io/xjh2v/.
