# Supplementary material for: Temporal attention causes systematic biases in visual confidence
Source: Sci Rep. 2019 Aug 12;9:11622. doi: 10.1038/s41598-019-48063-x (PMC6690997; doi:10.1038/s41598-019-48063-x)
Supplement: Supplementary file 1 — Supplementary Material [file 41598_2019_48063_MOESM1_ESM.pdf]

Temporal attention causes systematic biases in visual confidence

Supplementary Material

Authors: Samuel Recht (1)*, Pascal Mamassian (1), Vincent de Gardelle (2)

Affiliations:

(1) Laboratoire des systèmes perceptifs, Département d'études cognitives, École normale supérieure, PSL University, CNRS, 75005 Paris, France

(2) CNRS and Paris School of Economics, Paris, France

*Corresponding author: samuel.recht@gmail.com

Index

Experiment 1 3
- Position-based metacognition 3
- Delay in attentional selection 3
- Order reversals between T1 and T2 4

Experiment 2: a replication with lowered metacognitive load 6
- Material & methods 6
  - Participants 6
  - Apparatus and stimuli 6
- Analysis 6
- Results 7
  - T1: Distribution of reports 7
  - T1: Probability of report and confidence are correlated 7
  - T2: Confidence tracks the Attentional Blink but not lag-1 sparing 8
  - T2: Confidence in correct responses vs. errors 10
  - T2: Probability of report and confidence are correlated 10
  - T2: Delay in temporal selection and confidence 12
  - Order reversal between T1 and T2 13

Descriptive model for attention and confidence 14

References 20

Experiment 1

T1: Position-based metacognition

In a finer analysis, we tested whether participants' confidence could discriminate between different errors across different serial positions, not just between correct and incorrect responses. Excluding correct T1 responses, we found that a regression model with position effect and lag outperformed the null model without the position for predicting confidence ($\chi^2(3)=101.2$, $p_{RAND}<0.001$), with no significant interaction between position and lag ($\chi^2(12)=7.99$, $p_{RAND}=0.78$). Participants are thus sensitive to the difference between various position errors, even if this distinction is irrelevant to succeed in the present task.

T2: Delay in attentional selection

To analyze the delay in selection and confidence following reorienting of attention to T2, we calculated the average position of the reported item relative to the target position, in an 11-items window centered on the target position. This measure, called the "center of mass" (Goodbourn et al., 2016; Vul, Nieuwenstein, & Kanwisher, 2008) is positive when a delay occurs in item selection. Figure S1 illustrates the average center of mass across participants, separately for each lag, and shows that T2 item selection is delayed specifically after the Attentional Blink (at lags 6 and 9), replicating previous findings (Goodbourn et al., 2016; Vul, Nieuwenstein, et al., 2008). A model comparison approach confirmed that including the lag as a predictor for the center of mass significantly outperformed the null model ($\chi^2(4)=56.9$, $p_{RAND}<0.001$). Bonferroni-corrected t-tests (alpha=0.05/5) confirmed a significant effect at lag-2 ($t(30)=-3.3$, $p=0.002$), lag-6 ($T(30)=506$, $p<0.001$) and lag-9 ($T(30)=527$, $p<0.001$), but not for lag 1 and 3 (all $p>0.6$). The non-linearity observed from lags 1 to 3 should be considered with caution: it could reflect both the interaction with T1 attentional episode (Goodbourn et al., 2016) and the bi-modality of lag-3 reports distribution (see Fig. 4A). A similar analysis on T1 confirmed a significant effect of lag on the center of mass as well ($\chi^2(4)=19.4$, $p_{RAND}<0.001$). This positive center of mass for T1 was not necessary predicted by the literature (Goodbourn et al., 2016; Vul, Hanus, & Kanwisher, 2008; Vul, Nieuwenstein, & Kanwisher, 2008) although some datasets show a similar tendency (see e.g. Fig. S3 in the Supplementary Material of Goodbourn et al., 2016 and in particular the distribution of T1 latency for the "Western", "Berkeley", and "Sydney words" datasets, as well as estimated

delays in Martini, 2012). Interestingly, this delay disappeared in our replication with lowered metacognitive load (Exp. 2). The hypothesis that the observed T1 delay is the effect of (meta)cognitive load on selection would require further investigations. This positive delay, however, did not affected confidence (see below).

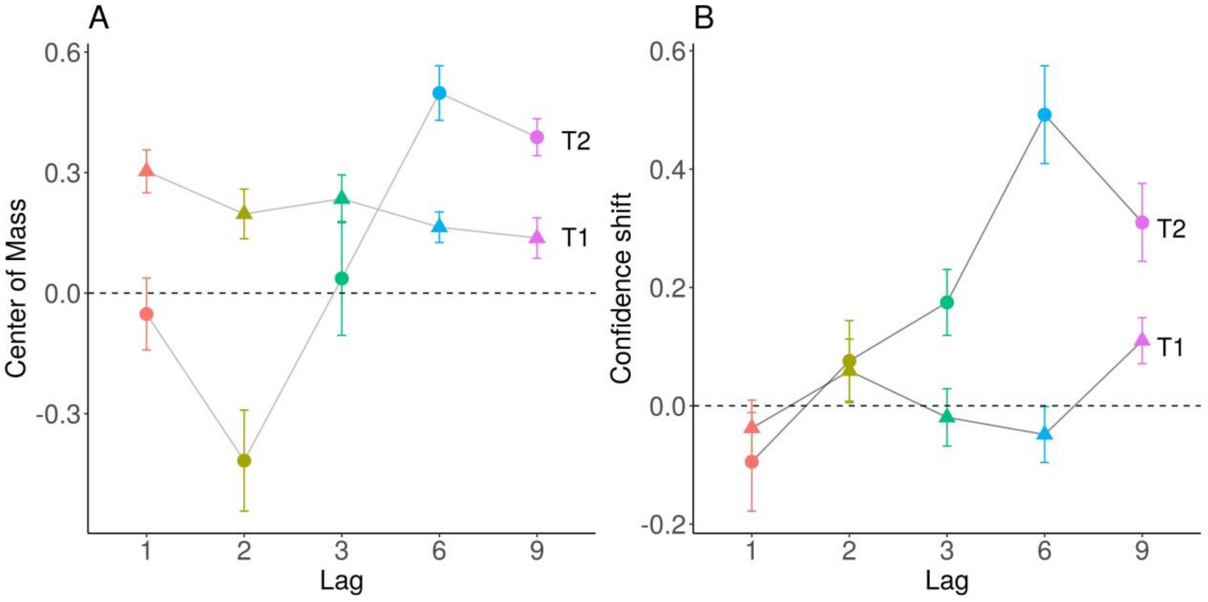

Figure S1: Delay is temporal selection. (A) The average center of mass for T1 (rectangles) and T2 (dots) as a function of lag. T2 center of mass is specifically delayed for lag-6 and lag-9. For T1, selection is slightly delayed but this remains stable across lags. (B) The confidence shift, which is the difference in average confidence between post-target and pre-target errors for T1 (triangles) and T2 (dots). A positive value corresponds to higher confidence for post-target errors, that is, a shift of the confidence peak towards more delayed items. Error bars represent standard error of the mean across participants.

Order reversals between T1 and T2

Order reversals occur at lag-1 when participants report both T1 and T2 but in the reverse order. In our data, order reversals occurred on average in 7.68% (SE ± 4.71%) of lag-1 trials. For comparison, correct report of both T1 and T2 in the correct order occurred in 12% (SE ± 5.77%) of lag-1 trials. To evaluate whether participants were aware of such reversals, the confidence between trials in which both T1 and T2 were correctly reported was compared to the confidence in reversed trials. One participant was discarded from this analysis due to no order reversal trial. No difference in confidence was found between these two types of trials,

neither for T1 ($t(30)=1.07$, $p=0.29$) nor for T2 ($t(30)=1.20$, $p=0.24$). Thus, it seems that participants were not specifically aware of the occurrence or non-occurrence of a reversal on a trial-by-trial basis. However, it is still possible that participants could be aware of the possibility of order reversals at lag-1 relative to longer lags, and that being aware of this possibility would be responsible for the lag-1 under-confidence.

Experiment 2: a replication with lowered metacognitive load

Material & methods

Participants

35 adult volunteers were recruited from the Laboratoire d'Economie Expérimentale de Paris (LEEP) pool of participants (M ± SD = 24.5 ± 3.06 years old, 18 females). They all provided informed written consent prior to the experiment. One observer was discarded for not finishing the experimental session, and 6 participants were removed because of extremely small accuracy rate for target 1 or 2 (exclusion criterion: <10% accuracy), leaving 29 participants for analysis. Observers were paid a base sum (10 EUR) plus a bonus depending on their performance in the task (up to 10 EUR in addition). The average payoff was 14.89 EUR (SD = 2.09) for a single 1.5 hours session. The experimental procedure received approval from the Paris School of Economics (PSE) ethics review board and adhered to the principles of the Declaration of Helsinki.

Apparatus and stimuli

Identical apparatus, stimuli and parameters were used for both experiments. The only difference being that for Experiment 2, confidence judgments was required only for T1 on half of the 500 trials, and only for T2 on the other half. Participants were divided into two groups to control for possible order effects. Participants were left uninformed that they will have to estimate their confidence for the other target until the end of the first half of the experiment.

Analysis

For the following analyses, trials were grouped by confidence probe: one group of trials for T1 confidence (250 trials per participant) and one group of trials for T2 confidence (250 trials). Therefore, even when accuracy only was considered, the average concerns the subset of trials related to the target where confidence judgment was requested.

Results

T1: Distribution of reports

The results from Experiment 1 were successfully replicated, with a significant effect of lag on accuracy ($F(3.4, 5.134)=9.1$, $MSE=0.005$, $p<0.001$) and confidence ($F(1.65,46.17)=17.5$, $MSE=0.08$, $p<0.001$). We found that letters presented just before or just after the target were reported on 19% of the trials (18% in Exp. 1), which exceeded the guess rate of 1/26 that is about 4% (mean corrected for guess rate: 0.15, 95% CI=[0.13 0.17]; $t(28)=14.7$ $p<0.001$).

To quantify how report frequency depended on serial position, we focused on serial positions from 2 items before to 2 items after T1 (included) and tested how report frequency can be predicted from the lag, the position and their interaction as fixed effects. Including item position as a predictor outperformed a model without the position effect ($\chi^2(4)=565.0$, $p_{RAND}=0.002$). Including the interaction between lag and position did not improved the model over a model without the interaction ($\chi^2(16)=21.6$, $p_{RAND}=0.36$), contrary to Exp. 1.

T1: Probability of report and confidence are correlated

Similarly to Exp. 1, confidence was affected by item position ($\chi^2(4)=94.03$, $p_{RAND}=0.003$). Including the interaction between lag and position however did not improve the model ($\chi^2(16)=26.0$, $p_{RAND}=0.16$). Given that for T1 data, both report frequency (Fig. S2A) and confidence (Fig. S2B) were affected by position in similar manners, we directly evaluated the correlation between confidence and report frequency. To do so, for each participant we averaged confidence over lags, and correlated this average confidence to the report frequency across 5 report positions centered on target (including target's true position). The mean r coefficient was 0.71 across participants (95% CI=[0.59 0.83]; $t(27)=11.9$, $p_{RAND}<0.001$), replicating Exp. 1.

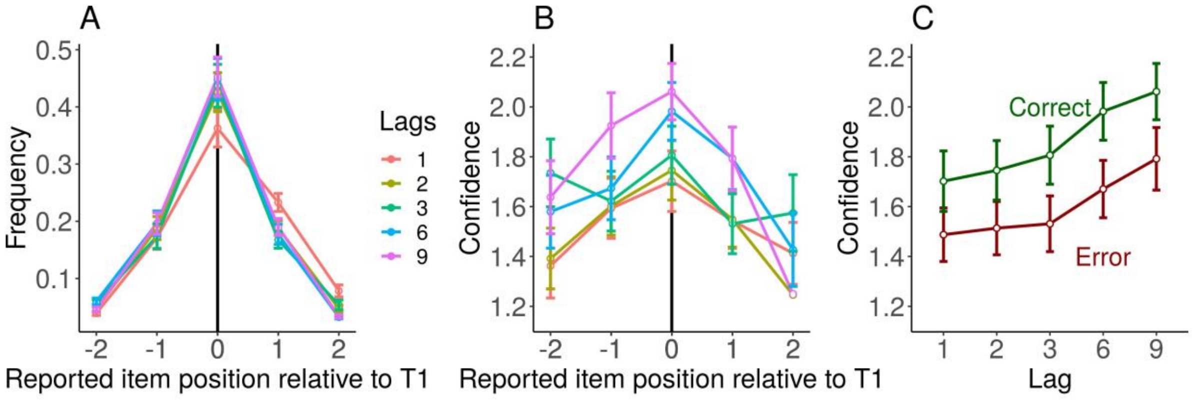

Figure S2: Reports and confidence about T1. (A) The frequency of report for item around target true position. (B) The corresponding average confidence per position. (C) The average confidence level for correct responses and errors, which provides an estimate of metacognition. Error bars represent standard error of the mean across participants.

Overall, T1 targets in Exp. 2 – as for Exp. 1 - were identified correctly 43% of the time. A main effect of trial type (error versus correct trial) was found ($F(1,28)=39.0$, $MSE=0.13$, $p<0.001$) but no interaction between lag and trial type ($F(3.598,100.737)=1.2$, $MSE=0.02$, $p=0.31$), confirming that participants had stable error-based metacognition for T1. Participants therefore gave higher confidence to correct than to incorrect T1 responses.

T2: Confidence tracks the Attentional Blink but not lag-1 sparing

Overall, 22% of T2 reports were correct when T1 was correctly reported. Figure S3A shows T2 accuracy and confidence for the different T1-T2 lags. As expected, the accuracy of T2 reports (i.e. in green) was affected by the lag between T1 and T2 ($F(2.9,81.13)=41.4$, $MSE=0.03$, $p<0.001$). In particular, the drop for lag 2 and lag 3 relative to longer lags (2-3 vs. 6-9: $T(28)=7$, $p<0.001$) indicated a classical Attentional Blink effect. Confidence was also affected by lag ($F(2.59,72.42) = 37.2$, $MSE=0.10$, $p<0.001$) and dropped for lags 2-3 relative to longer lags (2-3 vs. 6-9: $T(28)=0$, $p<0.001$), paralleling the drop observed for accuracy. Thus, participants seem able to acknowledge the drop of performance during the Attentional Blink that occurs at lags 2-3, in a similar manner as for Exp. 1.

Participants' confidence, similar to Exp. 1, seemed blind to lag-1 sparing. Indeed, the lag-1 sparing effect was also found in our data: T2 accuracy was spared when T2 was presented

immediately after T1. Accuracy at lag-1 was much higher than during the blink period (1 vs. 2-3: $T(28)=378$, $p<0.001$) and was in fact indistinguishable from accuracy at long lags (1 vs. 6-9: $T(28)=238$, $p=0.67$). By contrast, confidence was as low at lag-1 as it was for lag 2-3 ($T(28)=160$, $p=0.70$) and much lower than confidence at long lags (1 vs. 6-9: $T(28)=9$, $p<0.001$). All these results were fully coherent with what was found in Exp. 1.

Figure S3B shows confidence and accuracy at lag-1, in the lag-3-to-9 space, where lag-3 and lag-9 have (0,0) and (1,1) coordinates, respectively. Most participants are located below the diagonal, suggesting that they are less confident at lag-1 than what would be expected given their accuracy level at lag-1. This lag-1 under-confidence, calculated as the average difference between predicted and observed lag-1 confidence, was significant at the group level ($T(28)=325$, $p<0.001$, alpha=0.05/3). To confirm that this linear approach could nonetheless be used to predict confidence at another lag, we applied the same analysis to lag-2 and lag-6. The difference was significant neither for lag-2 ($t(28)=248$, $p=0.7$, alpha=0.05/3) nor for lag-6 ($t(28)=0.13$, $p=0.9$, alpha=0.05/3). These results suggest that probing confidence only for T2 did not alter the pattern found in Experiment 1.

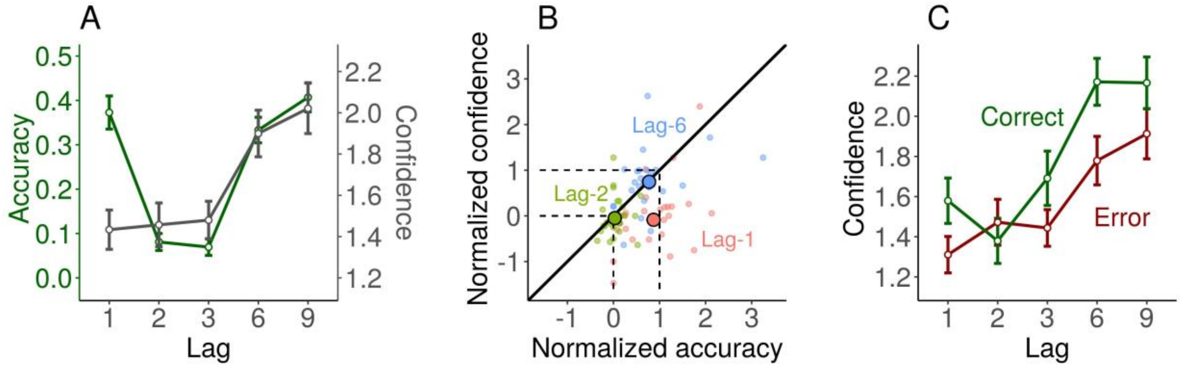

Figure S3: Attentional Blink and early confidence bias under lowered metacognitive load. (A) T2 average accuracy (in green) and confidence (in grey) as a function of the lag between T1 and T2. (B) The systematic under-confidence occurring at Lag-1 (see Fig. 3B) was also found in Experiment 2. Each point is a participant. (C) The average confidence level for correct T2 reports and errors, for each lag.

T2: Confidence in correct responses vs. errors

Because some participants had no correct answers during the Attentional Blink, only half of participants were considered here (N=14). As can be seen from Figure S3C, participants overall expressed higher confidence when they were correct relative to their errors, with a main effect of trial type (error vs correct, $F(1,13)=16.5$, $MSE=0.10$, $p=0.001$) and a main effect of lag ($F(3.1,40.7)= 21.8$ $MSE=0.16$, $p<0.001$), but no interaction ($F(1.9,24.1)=1.5$, $MSE=0.24$, $p=0.2$). This difference between Exp 1 and Exp 2 might relate to the difference in samples (250 vs 500) and the low number of participants in the present analysis (N=14).

T2: Probability of report and confidence are correlated

The similarity between confidence and report frequency was tested by looking at their correlation across lags for 5 positions centered on T2, but contrary to T1, the correlation was not reaching significance (Mean r coefficient: 0.55, 95% CI=[0.38 0.73]; $t(28)=6.5$, $p_{RAND}=0.06$), as shown on Figure S4D. Figure S4C plots the regression on one representative participant for illustrative purpose. The smaller correlation found in Exp. 2 compared to Exp. 1 might be the result of the reduced number of samples (half of Exp.1 samples for T2 confidence).

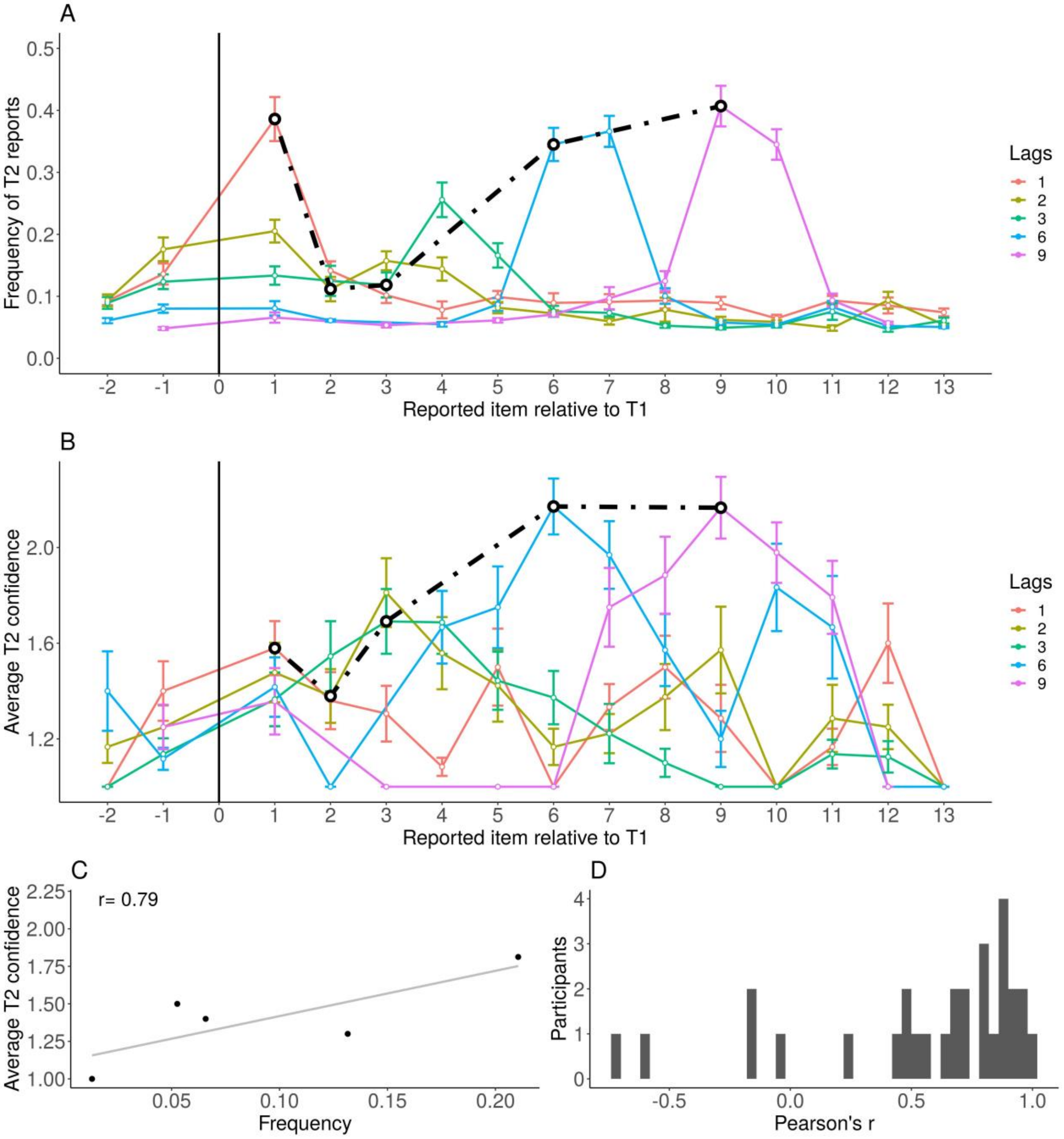

Figure S4: Reports and confidence about T2. (A) The frequency of T2 reports as a function of the position of the reported item relative to T1, for each lag. Note that T1 position has no value, given that only trials in which T1 is correctly reported were considered here (hence T2 reports cannot correspond to T1 position). The black line connects the points corresponding to accurate T2 reports. (B) Confidence of the T2 reports, as a function of the position of the reported item relative to T1, for each lag. The black line connects the points corresponding to accurate T2 reports. Error bars represent standard error of the mean across participants. (C) Regression between frequency and confidence with 5 positions centered on T2, collapsed across lags, for a representative participant. (D) Histogram of the correlation coefficients for all the participants.

T2: Delay in temporal selection and confidence

As for Exp. 1, items appearing just before or just after the T2 were more likely to be reported than chance (17%, with a 95% CI=[0.15 0.18]; vs. chance level at 4%: $t(28)=19.0$, $p<0.001$). Hence, errors were not random guesses but samples that are close to the actual T2 target. A model comparison approach confirmed that including the lag as a predictor for the center of mass significantly outperformed the null model for T2 ($\chi^2(4)=18.4$, $p_{RAND}<0.001$). Replicating Exp. 1, selection appears to be systematically too late for lags 6 and 9 (Fig. S5A). Bonferroni-corrected t-tests (alpha=0.05/5) confirmed an effect of lag on the center of mass for lag-6 ($t(28)=5$, $p<0.001$) and lag-9 ($t(28)=5.2$, $p<0.001$), but not for lag 1, 2 and 3 (all $p>0.15$).

For T1, the effect of lag on the center of mass was also significant ($\chi^2(4)=24.4$, $p_{RAND}<0.001$), but Bonferroni-corrected t-tests (alpha=0.05/5) confirmed that it was specifically driven by lag-1 ($t(28)=3.2$, $p<0.001$), but not by other lags (all $p>0.3$). This lag-1 effect on T1 selection delay could be resulting from order reversals (see below).

To analyze confidence, a model comparison approach confirmed that including the pre/post-target factor (or “shift”) as a predictor for average confidence significantly outperformed the null model ($\chi^2(1)=18.3$, $p_{RAND}<0.001$). The interaction between lag and shift was, however, not significant ($\chi^2(4)=6.6$, $p_{RAND}=0.08$). In other words, confidence is oblivious to the delays induced by the Attentional Blink and biased towards items selected later. A reduced metacognitive load in Exp. 2 did not enhance delay introspection (Fig. S5B). For comparison, we found no effect of shift on confidence for T1 ($\chi^2(1)=0.3$, $p_{RAND}=0.5$).

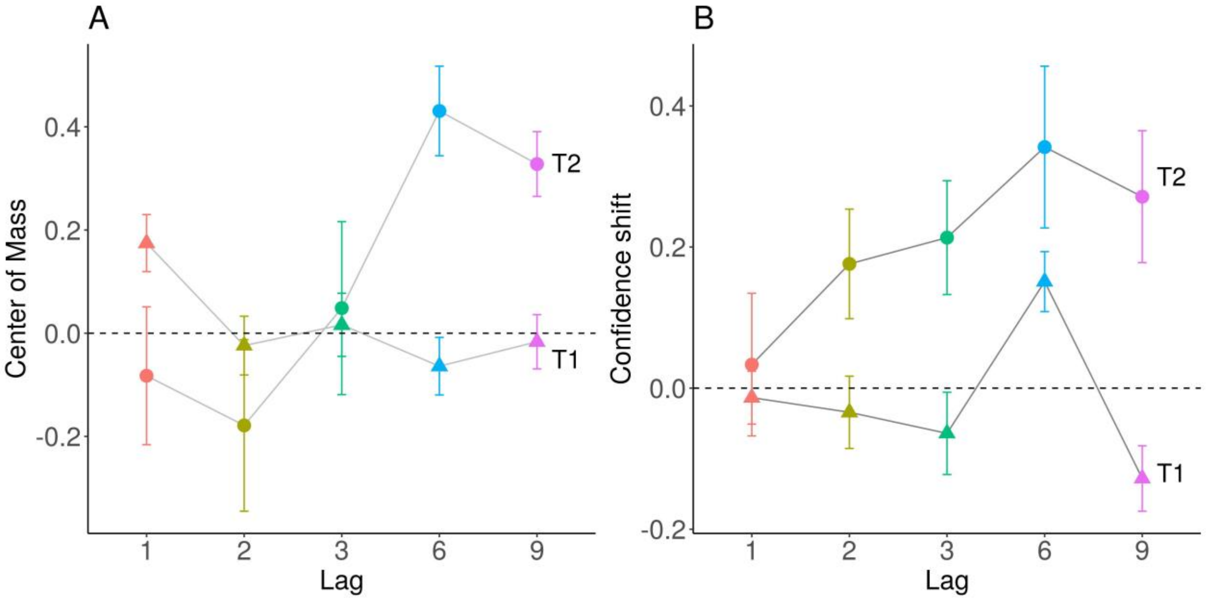

Figure S5: Confidence does not correct for attentional delay. (A) The average center of mass for T1 (rectangles) and T2 (dots) as a function of lag. Note the delay in T2 selection following lag-3. (B) The confidence shift, which is the difference in average confidence between post-target and pre-target errors for T1 (triangles) and T2 (dots). A positive value corresponds to higher confidence for post-target errors, that is, a shift of the confidence peak towards more delayed items. Error bars represent standard error of the mean across participants.

Order reversal between T1 and T2

In Exp.2, order reversals occurred on average in 5.2% (SE ± 4.1%) of lag-1 trials. For comparison, correct report of both T1 and T2 in the correct order occurred in 13.2% (SE ± 6.3%) of lag-1 trials. To evaluate whether participants were aware of such reversals, the confidence between trials in which both T1 and T2 were correctly reported was compared to the confidence in reversed trials. Seven participants were discarded from the later analysis due to no order reversal trial for the T1 confidence block, and two participants were discarded for the T2 confidence block. No difference in confidence was found between these two types of trials for T1 ($t(21)=0.4$, $p=0.70$), or for T2 ($t(26)=2.2$, $p=0.04$), after Bonferroni correction ($alpha=0.05/2$ for testing T1 and T2). Thus, it seems that when metacognitive load is reduced, participants were not more able to notice the occurrence or non-occurrence of a reversal on a trial-by-trial basis.

Descriptive model for attention and confidence

Here, we propose a simple implementation of a single target selection model inspired by the Attentional Gating Model (Reeves & Sperling, 1986), that could produce the relation between confidence and report frequency found in our data. The model has 3 components: a sensory stage, an attentional modulation, and a decision stage.

The sensory stage consists in a set of letter detectors or channels. Each channel has a preferred letter and when this letter is presented on the screen the channel is activated for a short period of time (Eq. 1). The activity s_c of each channel c at the sensory stage is defined as a Gaussian function of time t, with parameters μ representing the time at which the letter is presented, and τ the duration of the channel's response.

$$s_c(t) = \frac{1}{\tau\sqrt{2\pi}}\, e^{-\frac{1}{2}\left(\frac{t-\mu}{\tau}\right)^2} \quad \text{(Eq. 1)}$$

When a cue is presented on the screen, an attentional modulation is triggered that will amplify the activity of all channels for a brief period of time. The attentional modulation $a(t)$ involves a strength parameter A, and follows a Gaussian function of time (Eq. 2), with parameters μ_A and τ_A representing the center and spread in time of the attentional window. Note that the attentional modulation can be suppressed (e.g. for T2 at lag 3), which will be captured by the strength parameter A being reduced. This attentional modulation can also be delayed relative to the true position of the cue, which will be represented by the parameter μ_A.

$$a(t) = A\, \frac{1}{\tau_A\sqrt{2\pi}}\, e^{-\frac{1}{2}\left(\frac{t-\mu_A}{\tau_A}\right)^2} \quad \text{(Eq. 2)}$$

At the end of the trial, the resulting activity of the channel c (noted y_c) is the cumulated response of channel c over time, corrupted by normally distributed noise with standard deviation σ (Eq. 3).

$$y_c = \int_0^T s_c(t)\, a(t)dt + \varepsilon, \varepsilon \sim N(0, \sigma) \quad \text{(Eq. 3)}$$

Finally, the response corresponds to the letter associated with the channel with maximal activity, and the confidence associated with this response corresponds to the activity of this channel.

$$response = argmax_c(y_c)$$
$$confidence = max_c(y_c)$$

We simulated this process independently for T1 and for T2 at different lags. The duration of the sensory response and attentional boost, and the noise at the decision stage were kept constant across simulations ($\tau = 60$, $\tau_A = 80$, $\sigma=0.001$). The values for A and μ_A were defined separately for T1 ($A = .95$ and $\mu_A = 0$) and for T2 at the different lags (see Fig. S6 D and E), in order to roughly reproduce our behavioral results. For comparison with our actual data, the simulated confidence was binned into 3 values across all lags, separately for T1 and T2.

The R script for the model can be found on OSF: https://osf.io/xjh2v

Applying our analyses to these simulated data (see Fig. S7 – S10), we found that the model qualitatively produces the correlation between confidence and report frequency across positions (Fig S9), as anticipated. Unsurprisingly, this model was also able to reproduce the associated observations that confidence judgments for T2 are blind to delays in response selection (Fig. S10), and that they are higher for correct responses than for errors for T1 (Fig. S10) and for T2 (Fig. S10). We found also that as in our real data, the simulated T2 confidence was higher at longer lags (Fig. S9), although this presumably reflects the choice of parameter values across lags and should not be taken as a key aspect of our model. It is also clear that this simple model does not reproduce one main result of our study, which is the under-confidence found at lag-1 for T2. We anticipated that this model would not show such under-confidence at lag-1, as it implements a strong link between confidence and accuracy, and no factor that would affect lag-1 specifically. This result might require an additional component to the model.

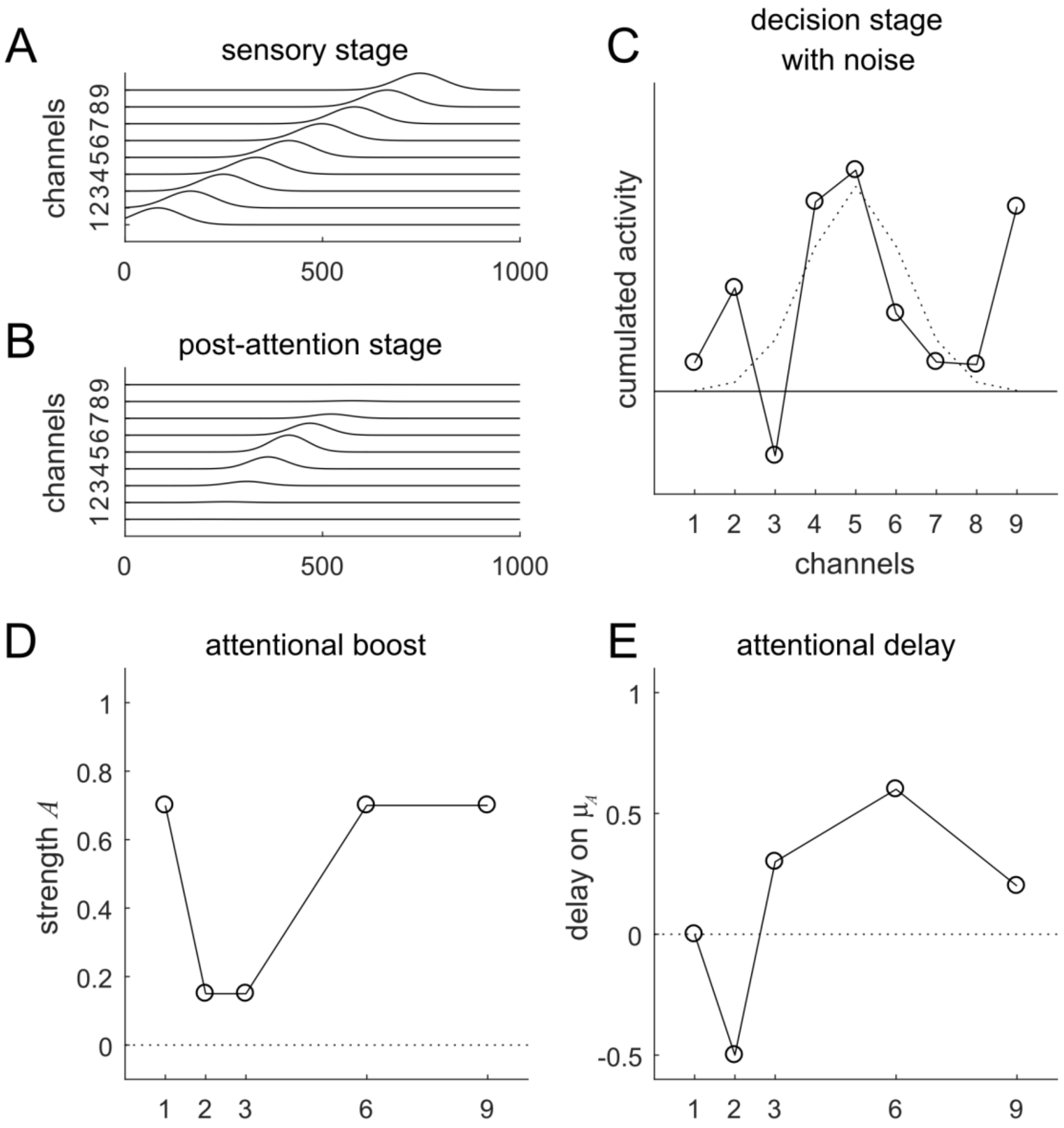

Figure S6: Illustration of the descriptive model. (A) Timecourse of activity in channels at the sensory stage. Only 9 channels are represented, which corresponded to the first 9 letters in the stream. (B) Timecourse of activity in channels after attentional modulation triggered at the 5th letter in the stream. (C) At the end of each trial, the activity in each channel (dotted line) is summed over time, and corrupted with additive noise (solid line). The identity of the best-responding channel, here channel 6, on a trial gives the response for that trial, and its activity gives the confidence. (D) The profile of attentional modulation across lags, used for our simulations of T2. (E) The profile of delay in attentional modulation across lags, used for our simulations of T2.

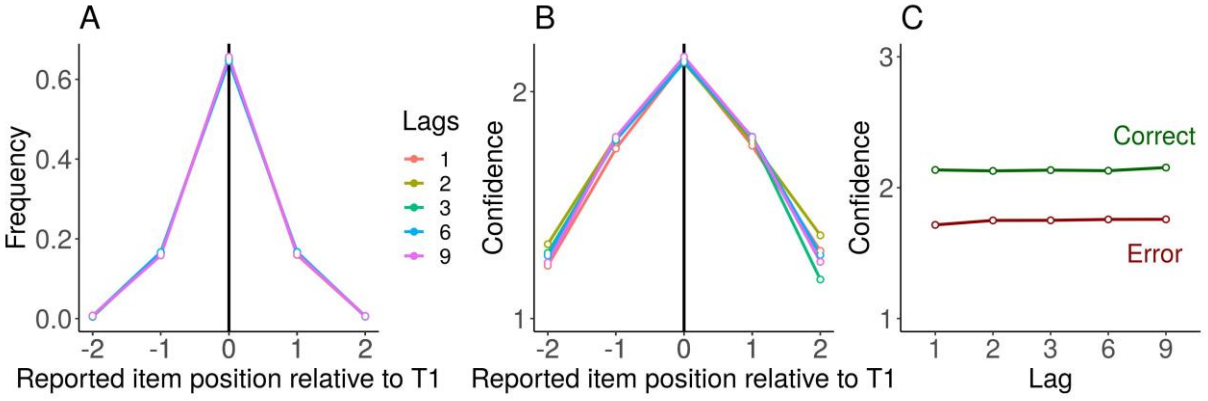

Figure S7: simulated reports and confidence about T1. (A) The frequency of report for item around target true position. (B) The corresponding average confidence per position. (C) The average confidence for correct and error trials.

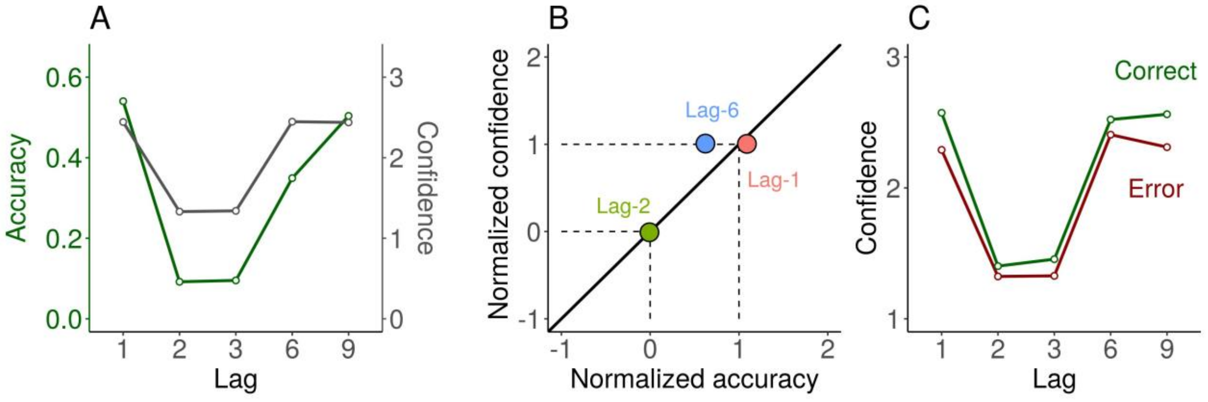

Figure S8: Attentional Blink but no early confidence bias for our simulated data. (A) T2 average accuracy (in green) and confidence (in grey) as a function of the lag between T1 and T2. (B) Simulated confidence and accuracy, normalized to the lag-3 to lag-9 interval. Note that the model does not produce the under-confidence at lag-1. (C) The average confidence level for correct responses and errors, for the different lags.

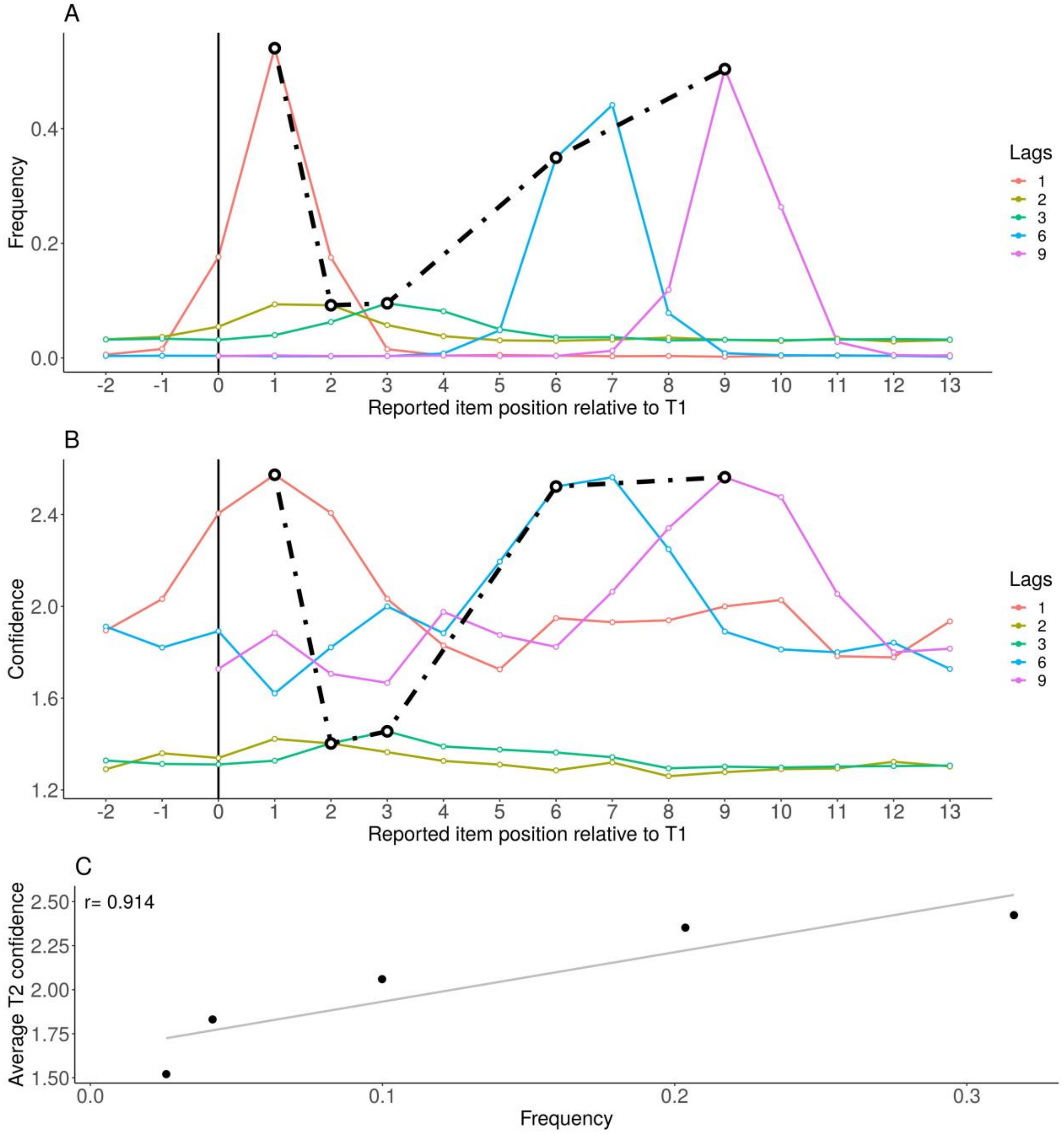

Figure S9: Simulated reports and confidence about T2. (A) The frequency of simulated T2 reports as a function of the position of the reported item relative to T1, for each lag. Note that T1 position has no value, given that only trials in which T1 is correctly reported were considered here (hence T2 reports cannot correspond to T1 position). The black line connects the points corresponding to accurate T2 reports. (B) Confidence of the simulated T2 reports, as a function of the position of the reported item relative to T1, for each lag. The black line connects the points corresponding to accurate T2 reports. (C) Regression between frequency and confidence with 5 positions centered on T2, collapsed across lags, for our simulation.

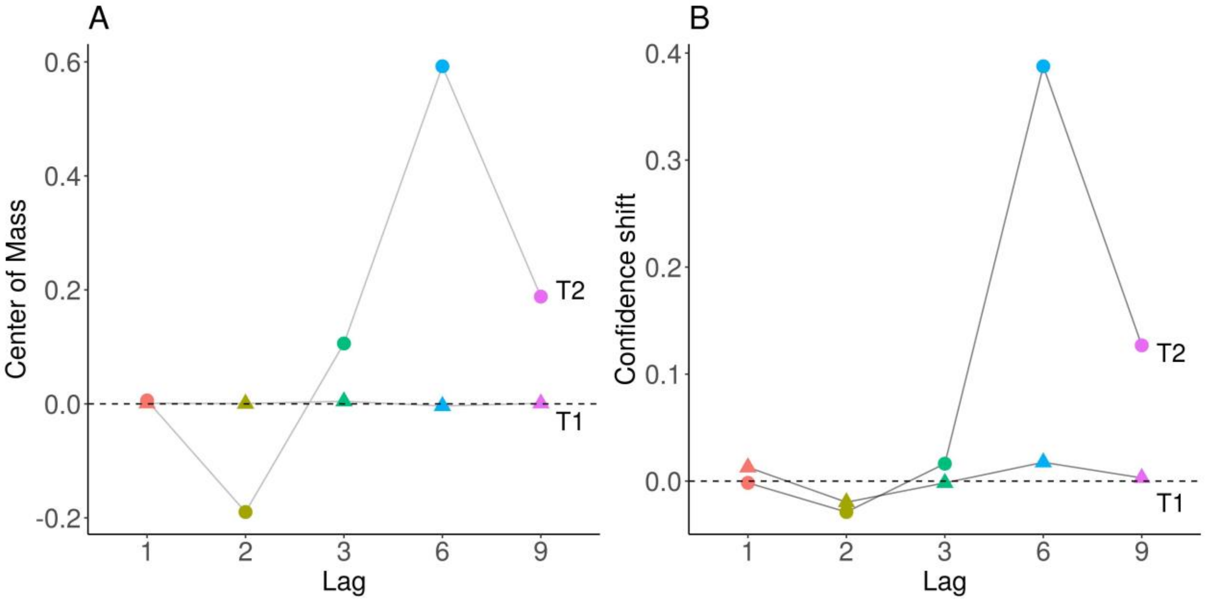

Figure S10: Simulated confidence does not correct for attentional delay. (A) The average center of mass for T1 (rectangles) and T2 (dots) as a function of lag, in our simulated data. Note the delay in selection following lag-3 for T2. (B) The confidence shift, which is the difference in average confidence between post-target and pre-target errors for T1 (triangles) and T2 (dots). A positive value corresponds to higher confidence for post-target errors, that is, a shift of the confidence peak towards more delayed items.

References

Goodbourn, P. T., Martini, P., Barnett-Cowan, M., Harris, I. M., Livesey, E. J., & Holcombe, A. O. (2016). Reconsidering Temporal Selection in the Attentional Blink. *Psychological Science*, *27*(8), 1146–1156. https://doi.org/10.1177/0956797616654131

Martini, P. (2012). Sources of bias and uncertainty in a visual temporal individuation task. *Attention, Perception, and Psychophysics*, *75*(1), 168–181. https://doi.org/10.3758/s13414-012-0384-y

Reeves, A., & Sperling, G. (1986). Attention gating in short-term visual memory. *Psychological Review*. https://doi.org/10.1037/0033-295X.93.2.180

Vul, E., Hanus, D., & Kanwisher, N. (2008). Delay of selective attention during the attentional blink. *Vision Research*, *48*(18), 1902–1909. https://doi.org/10.1016/j.visres.2008.06.009

Vul, E., Nieuwenstein, M., & Kanwisher, N. (2008). Temporal Selection is Suppressed, Delayed, and Diffused During the Attentional Blink. *Psychological Science*, *19*(1), 55–61. https://doi.org/10.1111/j.1467-9280.2008.02046.x
